# Fine structure of the aggregate silk nodules in the orb-web spider *Nephila clavata*

**DOI:** 10.1080/19768354.2018.1546227

**Published:** 2018-11-14

**Authors:** Myung-Jin Moon

**Affiliations:** Department of Biological Sciences, Dankook University, Cheonan, South Korea

**Keywords:** Aggregate gland, nodule, glue, *Nephila clavata*, silk, spider

## Abstract

Among the triad spinning units for capture thread producing system in the orb-web spiders, aqueous gluey substances are produced from two pairs of aggregate silk glands (ASG). Although biochemical analysis of glue substance is produced by way of their passage through the ASG, its structural modifications in the nodular area have been nearly neglected till now. This paper focused on identifying the fine structural characteristics of the aggregate nodules in the golden orb-web spider *Nephila clavata* using both of light and transmission electron microscopes. The ASG in *N. clavata* is composed of a multi-lobed secretory region and a thick excretory duct surrounded by large irregular nodules. Histological analysis of the nodules demonstrates that the nodule forming cells have extensive membrane-bound tubular system that is continuous with the surface membrane. In particular, the nodule forming cells contain numerous mitochondria and glycogen particles within their cytoplasms, and they are surrounded by the same sheath of thin connectives. As previously described, each gluey droplet is formed of a central glycoprotein mass surrounded by an aqueous covering components, the nodular organization in *N. clavata* indicates that the extensive membrane system is thought to have a function for gluey silk production in spider. The results of this study also strengthen the premise that spider glues are made of glycoproteins, and the aggregate nodule functions as a key component for the spider web glue production.

## Introduction

Capture threads in spiders are basically distinguished into two types with respect to the properties whose suitability for holding prey is based upon either a covering with a viscous glue (gluey capture threads) or a covering with extremely fine fibrils (cribellar capture threads) (Peters 1987). The gluey capture threads of the orb-web spiders in the vast family Araneidae and their relatives are produced by a triad of spigots on the posterior spinnerets (Foelix 2010). A pair of flagelliform glands produces supporting axial fibers, and two pairs of aggregate glands coat these fibers with an aqueous solution that quickly forms into sticky droplets (Vollrath et al. 1990; Peters and Kovoor 1991).

Although it is the flagelliform axial fibers that provide the primary tensile mechanics of the gluey capture threads (Opell and Bond 2000; Becker et al. 2003; Blackledge et al. 2005; Park and Moon 2014), the aggregate silk secretions make capture threads sticky and can modulate the mechanics of the axial core fibers (Opell and Hendricks 2010; Townley and Tillinghast 2013). Therefore, the aggregate silk coating of viscid threads spontaneously forms droplets as it is spun (Vollrath et al. 1990; Vollrath and Tillinghast 1991; Townley et al. 2006), and also allows spiders to achieve a greater stickiness per unit area in their webs (Opell 2002). Furthermore, viscid silk has reduced UV reflectance and this decrease in visibility may make webs more difficult for insects to avoid (Craig et al. 1994).

It has been generally known that the components of the viscous threads are composed of organic low-molecular-weight compounds (Fischer and Brander 1960), inorganic ions (Schildknecht et al. 1972), a phosphorylated glycoprotein (Tillinghast 1981), lipids (Peters and Kovoor 1991). The glycoprotein granules that coalesce inside each droplet contribute to thread adhesion (Vollrath and Tillinghast 1991) and the hydrophilic compounds in the surrounding fluid attract atmospheric moisture to prevent droplets from drying (Opell et al. 2011).

Due to the strong and heavy-duty sticky characteristics, these gluey substances also have a great variety of industrial uses. Although it has repeatedly been reported that the gluey substances of the capture threads in the orb-web spiders are produced from the ASGs (Peters 1987; Moon and Kim 2005; Park and Moon 2014), the glandular aspects on the production of the viscid silk in the spiders are not fully understood until now. Moreover, the physiological significance of the aggregate nodules and their exact contribution to the web glue production have been nearly neglected except for brief studies (Moon and Kim 1989; Peters and Kovoor 1991). Therefore, we describe here the microstructural framework of the aggregate nodules that surround outer surface of the ASG duct and the fine structural aspects of the nodule forming cells which contribute to the production of aqueous gluey substances in the orb-web spider, *N. clavata*.

## Materials and methods

Adult females of the golden orb-web spider, *Nephila clavata* (Araneae: Nephilidae) were collected in a local area near the Cheonan campus of Dankook University, Chungnam, Korea. All spiders were maintained under ambient conditions with natural lighting in enclosures comprising a wooden frame (height × length × width = 50 × 50 × 10 cm) with glass panels on the front and back, and fed insect larvae and water daily.

For histologic preparation, specimens were anesthetized with CO_2_ and dissected under a dissecting light microscope in a drop of spider Ringer's solution consisting of 160 mM NaCl, 7.5 mM KCl, 4 mM CaCl_2_, 1 mM MgCl_2_, 4 mM NaHCO_3_, 20 mM glucose, pH 7.4 (Moon and Tillinghast 2013). Both of spinnerets and silk glands were gently removed and fixed in alcoholic Bouin’s solution, and dehydrated through an ethanol series from 30% to l00% (30 min at each concentration, with one repeat at 100% ethanol). After dehydration, the specimens were transferred to xylene for clearing at room temperature to 60°C, and they were embedded with Paraplast embedding medium (Fisher Scientific Co., Pittsburgh, Pa, USA) at 60°C. The sections were cut with a thickness of approximately 5 µm using a rotary microtome, Histocut 820-II (Reichert-Jung, Germany) and they were stained with hematoxylin and eosin (H & E) solutions.

For scanning electron microscopic (SEM) experiment, the specimens were prefixed in a mixture of 2% paraformaldehyde and 2.5% glutaraldehyde buffered with 0.1 M phosphate buffer at pH 7.4. Postfixation was performed with 1% osmium tetroxide in the same buffer and washed several times in 0.1 M phosphate buffer. Following fixation, the specimens were carefully dehydrated in ascending concentrations of ethanol from 30% to l00%, and then either critical point-dried or transferred to hexamethyldisilazane (HMDS) for air-dry. All samples were coated to a thickness of approximately 20 nm with gold-palladium alloy using a sputter coater and examined on a Hitachi S-4300 (Hitachi Co., Japan) field emission scanning electron microscopy (FESEM) operated with an accelerating voltage of 5–15 kV.

For transmission electron microscopic (TEM) experiment, both spinnerets and silk glands were fixed and dehydrated according to the same protocol for SEM experiment. The specimens were then embedded in Poly/Bed 812-Araldite medium (Polysciences Inc., Warrington, PA, USA) via propylene oxide. Semi-thin sections, 0.5–1.0 µm thick, were obtained using an LKB Ultratome V (LKB, Stockholm, Sweden) and were stained with 1% toluidine blue (dissolved in 1% borax). Microscopic images were photographed using Zeiss Axiophot microscope (Carl Zeiss, Jena, Germany) coupled with a Motic digital imaging system (Motic Instruments Inc., Richmond, Canada). Ultrathin sections were obtained using an Ultra 45° diamond knife (Diatome, Hartfield, PA, USA), and were double stained with uranyl acetate, followed by lead citrate. After these treatments, the sections were examined with a JEM 100 CX-II transmission electron microscope (JEOL Ltd., Tokyo, Japan) at 80 kV.

## Results

**Figure 1.**
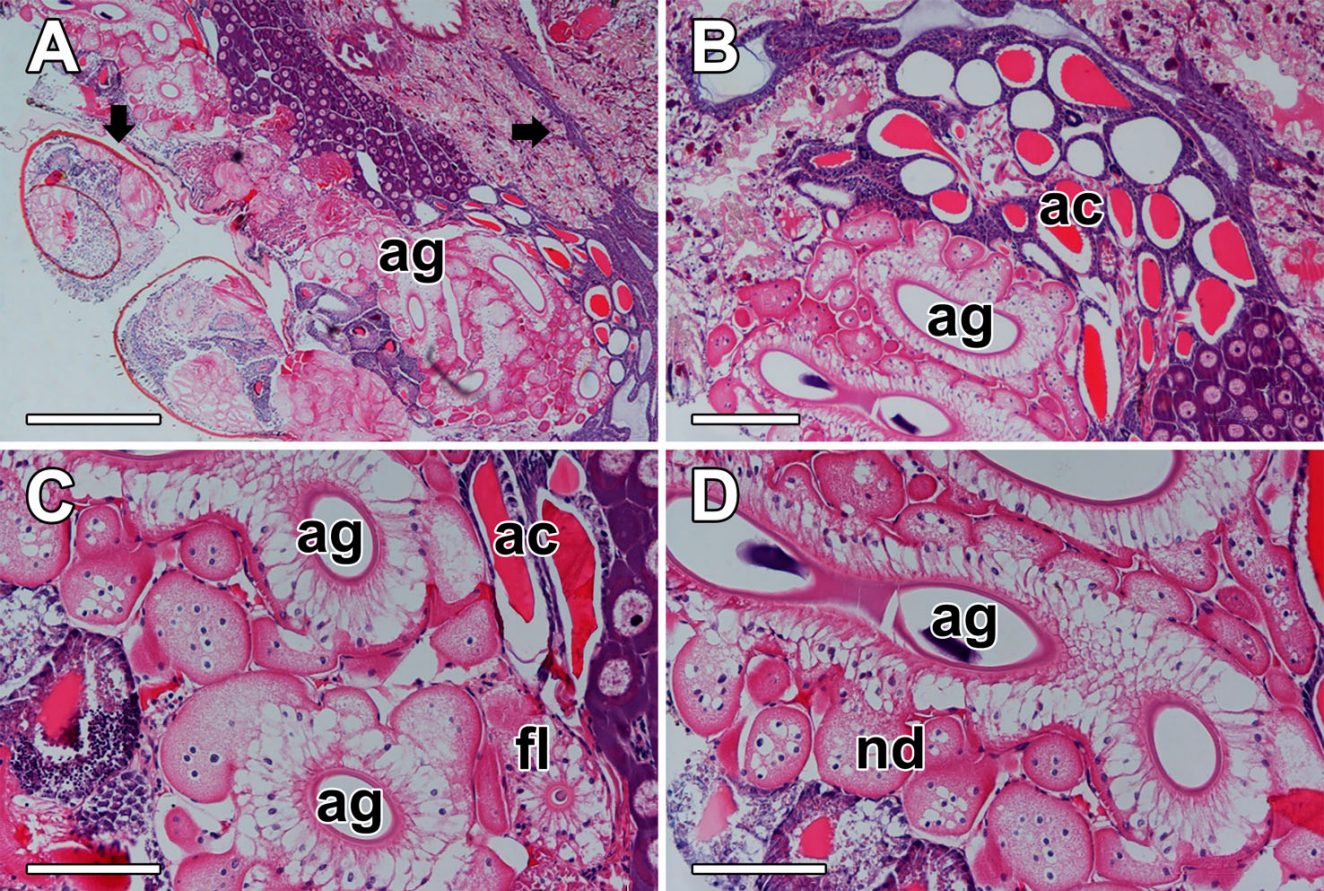
Photo micrographs of the opisthosoma in the spider *N. clavata*. (A and B) Sticky capture threads are produced from a pair of triad silk glands which composed of a flagelliform gland (fl) and two aggregate glands (ag). (C and D) Excretory ducts of the aggregate glands are surrounded by several large nodules (nd) in the posterior spinnerets. ac: Aciniform gland. Scale bars indicate 500 μm (A), 200 μm (B) and 100 μm (C and D), respectively.

Histologic preparation of the opisthosoma is shown that the sticky capture threads are produced from a pair of triad silk glands which composed of a flagelliform silk gland (FSG) and two ASGs (Figure 1(A,B)). Although the multi-lobed secretory regions of ASGs are widely distributed in the abdominal segment, their excretory ducts surrounded by several large nodules are just connected through the posterior spinnerets (Figure 1(C,D)).

**Figure 2.**
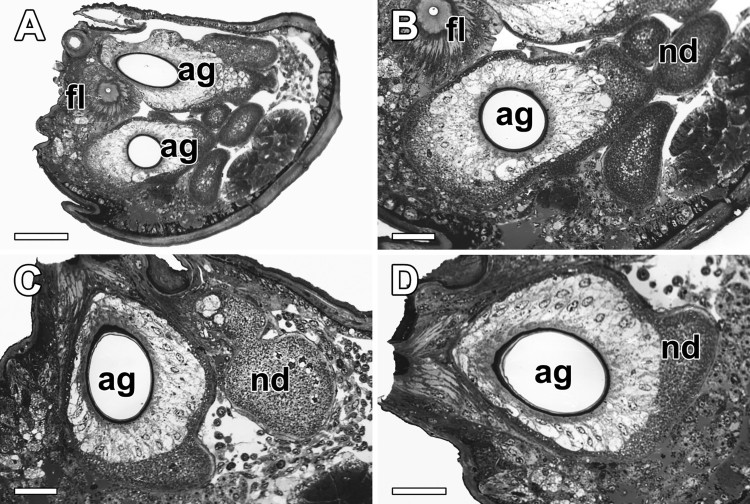
Photo micrographs of the posterior spinneret in *N. clavata*. (A and B) Excretory ducts of the central flagelliform gland (fl) and peripheral aggregate glands (ag) form a triad orientation along a single axis. (C and D) The ASG possesses thick excretory duct surrounded by large nodules (nd). Each duct is composed of the cuticle, epithelium, nodules and connectives. Scale bars indicate 100 μm (A) and 50 μm (B–D), respectively.

By the light microscopic observation embedded in the plastic EM-bed medium, the posterior spinneret in female spider is filled with four kinds of silk glands including FSG, ASGs, tubuliforms, and aciniforms. Excretory ducts of the FSG and ASG are arranged in a triad complex pattern so that the central FSG and peripheral ASGs form a linear orientation along a single axis (Figure 2(A)). Among them, the ASG which produce liquid gluey substances of the capture thread possesses thick excretory duct surrounded by large and irregular shaped nodules (Figure 2(B)). Distal duct of the ASG represents a homogeneous tube which is composed of the epithelial cells, the nodule forming cells, and the connective cells (Figure 2(C,D)).

**Figure 3.**
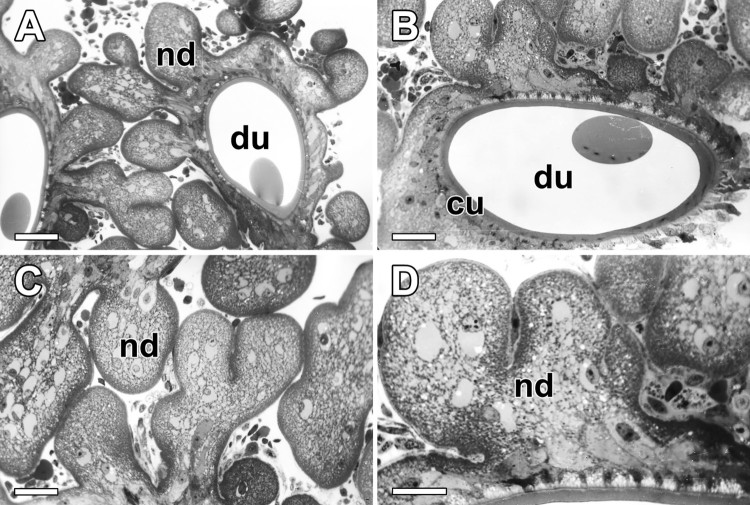
Photo micrographs of the nodules attached to duct of the ASG in *N. clavata*. (A and B) Numerous nodules (nd) are surrounded by the same sheath of thin connective tissues, and they are compactly aggregated along the outer surface of the excretory duct (du). (C and D) The nodules appear to be aggregated with solid or fluid-filled lumps. Maturity of the nodule can be perceived from the appearance of cytoplasmic inclusions. Scale bars indicate 20 μm (A), 10 μm (B and C), and 5 μm (D), respectively.

In the light microscope, gross morphology of the nodules and outer connectives are clearly visible (Figure 3(A)). The dimensions of these nodules are approximately 30 µm in average diameter and they are compactly aggregated along the outer surface of the excretory duct (Figure 3(B)). The nodules appear to be aggregated with solid or fluid-filled lumps. These numerous nodules are surrounded by the same sheath of thin connective tissues (Figure 3(C)). Cellular maturity of the nodule can be perceived from the appearance of cytoplasmic inclusions since secretory vesicles differ from cell to cell and can be representative of the secretory cycle (Figure 3(D)).

**Figure 4.**
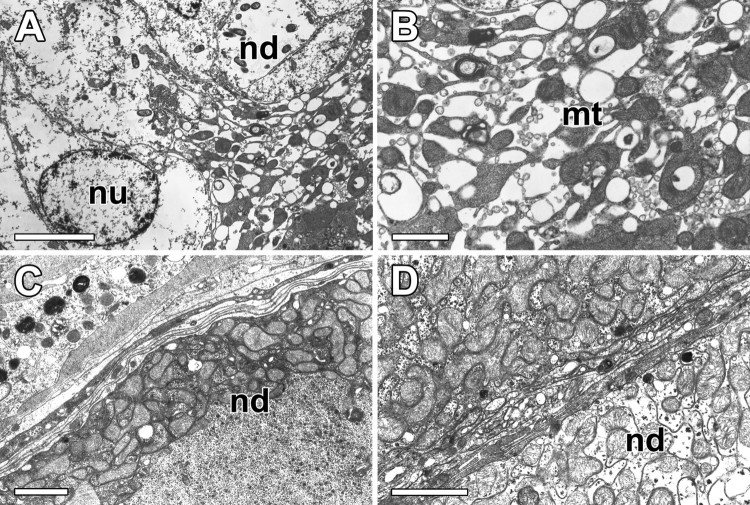
Transmission transmission electron micrographs of the ASG nodules in *N. clavata*. (A) Walls of the nodules are surrounded by extremely thin layers of plasma membrane. nu: nucleus. (B) Nodule forming cells are occupied by numerous mitochondria (mt) and homogeneous substances with high electron-opacity. (C) Mitochondria are scattered throughout cytoplasm of the nodules (nd), and the rest of cytoplasm is occupied by glycogen particles. (D) Each nodule has an invaginated cytoplasm which consists of densely packed mitochondria and other cellular organelles involved in protein synthesis and secretion. Scale bars indicate 20 μm (A), 5 μm (B) and 2 μm (C and D), respectively.

In the transmission electron microscopic observation, the nodules of the ASG is composed of large nodule forming cells approximately 25 µm in average diameter of nucleus. The walls of the nodules are extremely thin, consisting only of the basal plasma membrane, except where the transitional zone between duct and nodule (Figure 4(A)). The cytoplasmic processes of the nodule forming cells are surrounded by invaginations of the plasma membranes. The large nodule forming cells with more than 50% of their cytoplasm is occupied by mitochondria. The other cytoplasmic component in nodule is a homogeneous substance of glycogen particles with high electron-opacity (Figure 4(B)).

TEM examination of the nodule reveals that it is composed of numerous mitochondria and aggregates of glycogen particles with folded cytoplasmic processes. Mitochondria are scattered throughout the cytoplasm, and the rest of the cytoplasm is occupied largely by glycogen particles. The apical portion of the nodule is filled with secretory droplets which surrounded by a limiting membrane. Numerous multivesicular bodies which presumed to be the precursors of glue materials appeared in the cytoplasm of this nodule (Figure 4(C)). The basal infoldings form narrow compartments that interdigitate with similar processes of neighboring cells. Numerous mitochondria lie in the cytoplasm within the folds. The large nodules of the ASG have invaginated cytoplasms composed of large cells with an electron-lucent cytoplasm, which consists of densely packed mitochondria and other cellular organelles involved in protein synthesis and secretion (Figure 4(D)).

**Figure 5.**
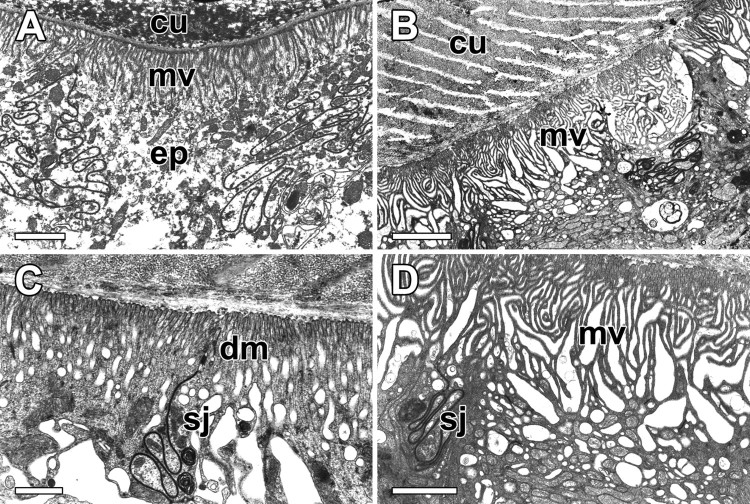
Transmission electron micrographs of the duct of the ASG in *N. clavata*. (A and B) Duct cuticles (cu) are composed of endocuticle and exocuticle. Around the apical surface of the epithelial cells (ep), thousands of microvilli (mv) form a brush border. (C and D) Specialized septate junctions (sj) and desmosomes (dm) are seen along the plasma membranes of the adjacent epithelial cells. Scale bars indicate 2 μm (A and B) and 1 μm (C and D).

The epithelial cells of the ASG duct are composed of cuboidal cells with moderate nuclei. The cuticles of the proximal duct near the secretory portion are composed of endocuticle and exocuticle (Figure 5(A)). On the apical surface of the epithelial cells, thousands of microvilli form a brush border structure which provides the cells with a higher surface area to volume ratio (Figure 5(B)). Specialized septate junctions and desmosomes are formed along the plasma membranes of the adjacent epithelial cells (Figure 5(C)). The lumen or apical border of the cells comprises a series of microvilli. The apices of the epithelial cells are fringed with short and irregular microvilli (Figure 5(D)).

**Figure 6.**
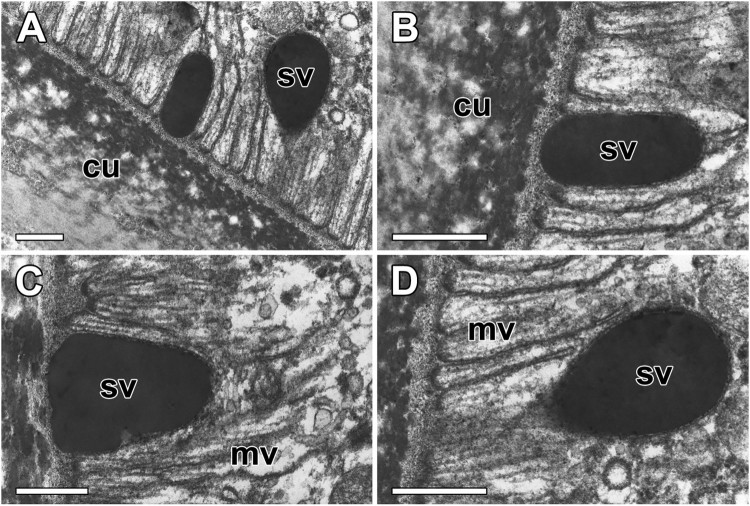
High magnification electron micrographs of the secretory materials in ASG in *N. clavata*. (A and B) secretory vesicles (sv) in epithelial cell appears as an almost spherical material with high electron density. (C and D) These vesicles are transported to cuticular layers (cu), and they are fused with several others subsequently to form amorphous electron dense deposits of endocuticle. mv: microvilli. All scale bar indicates 0.5 μm.

The secretory materials accumulate inner duct wall have a unique microstructure with electron dense vesicles. These electron dense vesicles are densely packed and remain close to each other without fusion (Figure 6(A)). The mature secretory product in epithelial cell appears as almost spherical vesicles range in size from 0.5–1.0 µm and resemble what has been described as protein droplets (Figure 6(B)). These secretory vesicles originate in distended cisternae where they attached to the microvilli of the epithelial cells (Figure 6(C)). Vesicles are frequently seen being secreted into the lumen at the apical surface of the cells. These vesicles appeared to be transported to cuticular layers and aggregate with several others, forming amorphous electron dense deposits of endocuticle (Figure 6(D)).

## Discussion

The web glue that coat the sticky spirals of the capture threads in orb-web spiders is considered as one of the strongest and most effective biological glue (Vollrath et al. 1990; Opell and Hendricks 2010). The sticky or adhesive threads in *N. clavata* are produced by a pair of triad spigots which composed of an FSG and two ASGs on the posterior spinnerets as it has been previously shown in other orb-web spiders (Peters 1987; Peters and Kovoor 1991; Townley et al. 2006). However, the triad spigots in *N. clavata* are arranged in a specific pattern so that the central FSG and peripheral ASGs form a linear orientation along a single axis, characteristically.

The supporting fibers of viscous capture threads are covered by aqueous droplets that derive their stickiness from internal nodules. Although most of a substance from the ASG is consolidated into droplets, a sheath of aqueous material surrounds axial fibers in inter-droplet regions (Vollrath et al. 1990; Opell and Hendricks 2010). Moreover, the walls of the nodules are extremely thin, and the cytoplasmic processes of the nodule forming cells are occupied by numerous mitochondria and glycogen particles both involved in the generation of ASG nodules. Thus, this suggests that the organelles present in cytoplasm have some specific vital functions for web glue production.

The function of mitochondria is to produce and store energy (McBride et al. 2006), while endoplasmic reticulum facilitates synthesis and transport of protein, production of steroids, production as well as storage of glycogen (Lodish et al. 2003). In addition, glycogen is the most common form of glucose in animals and is especially abundant in cells as clusters or rosette of beta particles that resemble ribosomes, located near the smooth endoplasmic reticulum (Berg 2006). Glycogen is an important energy source of the cell, therefore it will be available on demand, the enzymes responsible for glycogenolysis degrade glycogen into individual molecules of glucose and can be utilized by multiple organs of the body (Lodish et al. 2003).

It has been reported that the adhesive droplets in the orb webs of araneoid spiders contain an aqueous solution of organic low-molecular-weight compounds (Fischer and Brander 1960; Vollrath et al. 1990; Opell 2002). In particular, adhesive silk threads of ecribellate spiders are covered with a sticky aqueous layer containing organic molecules, salts, fatty acids and small glycoproteins which are produced in the ASG of spiders (Vollrath and Tillinghast 1991; Townley et al. 2006). Recent researches have clearly shown that the suspended droplets of *Larinioides cornutus* viscous threads were composed of three unique layers – a droplet’s aqueous covering, barely visible glycoprotein glue and anchoring granule regions (Opell and Hendricks 2010). Moreover, two glycoproteins expressed from opposite strand of the same DNA sequence are purified from the aqueous coat of the golden orb weaving spider, *N. clavipes* (Choresh et al. 2009).

These reports strengthen the premise that glycogen functions as a key component in spider web glue. It is known that the major component of the glue as microscopic nodules is made of a glycoprotein (Tillinghast 1981; Vollrath et al. 1990; Vollrath and Tillinghast 1991). Tillinghast et al. (1993) purified a glycoprotein from orb webs of the spider *Argiope aurantia* and showed its morphology and biochemical traits through their electron microscopic examination of the glycoprotein preparation. They found that the glycoprotein shares several characteristics with mammalian secretory mucins which exhibit considerable flexibility. Choresh et al. (2009) also demonstrated that the glycoproteins of the web glue of the spider *N. clavipes* are highly glycosylated and have a high proportion of charged amino acids in the nonrepetive region. Both traits of glycopretein are expected to act as sticky glue substances since highly glycosylated proteins, such as mucins are known to have adhesive traits (Opell and Hendricks 2010) and those of charged amino acids could aid in retention of water for sticky droplets (Opell et al. 2011).

Fine structural examination revealed the ASG duct in *N. clavata* is surrounded by nodules which appear to be aggregated with solid or fluid-filled lumps. In addition, modular maturity can be perceived from the appearance of cytoplasmic inclusions since secretory inclusions differ from cell to cell and can be representative of the secretory cycle. Although we do not know exactly what this material is, one possibility would be a glycogen or starch since most insoluble cytoplasmic inclusions or substances on the cytosol are made up of glycogen particles and function as energy storage area (Berg 2006).

TEM examination of the nodule clearly shown that the cytoplasmic processes of the nodule forming cells are surrounded by invaginations of the plasma membranes. Infoldings of the basolateral region of the plasma membrane are commonly found in cells engaged in the active transport of fluids and ions. These infoldings seem to increase the surface area available for transport. It has been noted that the increased concentration of mitochondria is accompanied by a gradual disappearance from the cytoplasm of other cytoplasmic membrane systems and loss of plasma membrane specializations (Tandler et al. 1970).

Moreover, each nodule forming cell was demonstrated to have mitochondria with more than 50% of its total cytoplasmic volume. This excessive content of mitochondria showed a morphological sign suggesting the massive release of substances through its luminal surface into the duct space. Since mitochondria can be considered the power generators of the cell, providing the necessary energy for ion pumps located in the plasma membrane.

Tandler (1966) reported previously with electron microscopy that benign salivary gland oncocyte unusually contains high numbers of mitochondria. Furthermore, cytoplasmic granularity is due to the accumulation of mitochondria that may occupy up to 60% of the cytoplasm comparing to those of normal acinar cells which occupy only 5.2% (Chaushu et al. 1999). Recent electron microscopic observation of the oncocytes showed numerous tightly packed mitochondria of varying size and shape filling most of the cytoplasmic compartment (Arismendi-Morillo 2011).

This is consistent with the EM observations of the nodule forming cells in this research. In particular, the cells in aggregate nodule possess a very high proportion of mitochondrial contents and complex membrane systems for gluey silk production. It is likely that the mitochondria, when functioning as an energy provider during silk production, are involved in controlling the accumulation of secreted material into vesicles, and to permit the vesicle fusion and cycling necessary for the secretion of aqueous gluey substances.
